# (1*R*,2*R*,3*R*,4*R*,5*S*)-2,3-Bis[(2*S*′)-2-acet­oxy-2-phenyl­acet­oxy]-4-azido-1-[(2,4-dinitro­phen­yl)hydrazono­meth­yl]bicyclo­[3.1.0]hexa­ne

**DOI:** 10.1107/S1600536808000718

**Published:** 2008-01-16

**Authors:** Jing Li, Todd L. Lowary, Robert McDonald

**Affiliations:** a Alberta Ingenuity Centre for Carbohydrate Science, Department of Chemistry, University of Alberta, Edmonton, Alberta, Canada T6G 2G2; b X-ray Crystallography Laboratory, Department of Chemistry, University of Alberta, Edmonton, Alberta, Canada T6G 2G2

## Abstract

In the title compound, C_38_H_29_N_7_O_12_, the five-membered ring adopts an envelope conformation in which the ‘flap’ is *cis* to the cyclo­propane group. This conformation is similar to those of other bicyclo­[3.1.0]hexane analogues for which crystal structures have been reported. The absolute configuration of the stereogenic centers on the cyclo­pentane ring, as determined by comparison with the known configurations of the stereogenic centers in the (2*S*)-2-acet­oxy-2-phenyl­acet­oxy groups, is 1(*R*), 2(*R*), 3(*R*), 4(*R*) and 5(*S*). An intramolecular N—H⋯O hydrogen bond is present.

## Related literature

For the synthesis of mimetics of biologically important furan­oside rings, see: Callam & Lowary (2000 ▶); Callam & Lowary (2001 ▶); Callam *et al.* (2001 ▶); Centrone & Lowary (2002 ▶). For examples of the crystal structures of bicyclo­[3.1.0]hexane systems, see: Gurskaya *et al.* (1990 ▶, 1996 ▶); Gallucci *et al.* (2000 ▶); Garcia *et al.* (1992 ▶); Guthrie *et al.* (1981 ▶); Màrton-Merész *et al.* (1983 ▶); Biswas *et al.* (1996 ▶); Bai *et al.* (2004 ▶). For related literature, see: Li & Lowary (2008 ▶).
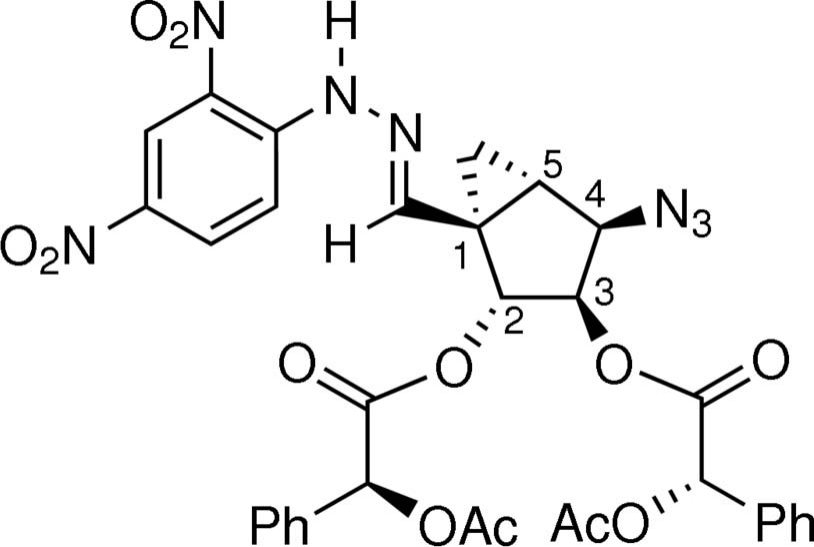


## Experimental

### 

#### Crystal data

C_33_H_29_N_7_O_12_
*M* *_r_* = 715.63
Monoclinic, 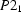

*a* = 6.8522 (11) Å
*b* = 17.747 (3) Å
*c* = 13.729 (2) Å
β = 99.006 (2)°
*V* = 1648.9 (5) Å^3^
*Z* = 2
Mo *K*α radiation
μ = 0.11 mm^−1^
*T* = 193 (2) K
0.63 × 0.56 × 0.04 mm

#### Data collection

Bruker SMART 1000 CCD area-detector/PLATFORM diffractometer
Absorption correction: multi-scan (*SADABS*; Bruker, 2003 ▶) *T*_min_ = 0.756, *T*_max_ = 0.996
13919 measured reflections
3905 independent reflections
3150 reflections with *I* > 2σ(*I*)
*R*_int_ = 0.040

#### Refinement

*R*[*F* ^2^ > 2σ(*F* ^2^)] = 0.043
*wR*(*F* ^2^) = 0.104
*S* = 1.04
3905 reflections
471 parameters
1 restraint
H-atom parameters constrained
Δρ_max_ = 0.20 e Å^−3^
Δρ_min_ = −0.23 e Å^−3^

### 

Data collection: *SMART* (Bruker, 2001 ▶); cell refinement: *SAINT* (Bruker, 2003 ▶); data reduction: *SAINT* (Bruker, 2003 ▶); program(s) used to solve structure: *SHELXS97* (Sheldrick, 2008 ▶); program(s) used to refine structure: *SHELXL97* (Sheldrick, 2008 ▶); molecular graphics: *SHELXTL* (Sheldrick, 2008 ▶); software used to prepare material for publication: *SHELXTL*.

## Supplementary Material

Crystal structure: contains datablocks global, III. DOI: 10.1107/S1600536808000718/bq2060sup1.cif

Structure factors: contains datablocks III. DOI: 10.1107/S1600536808000718/bq2060IIIsup2.hkl

Additional supplementary materials:  crystallographic information; 3D view; checkCIF report

## Figures and Tables

**Table 1:** Hydrogen-bond geometry (Å, °)

*D*—H⋯*A*	*D*—H	H⋯*A*	*D*⋯*A*	*D*—H⋯*A*
N11—H11N⋯O10	0.88	2.00	2.618 (3)	126

**Fig. 1.**
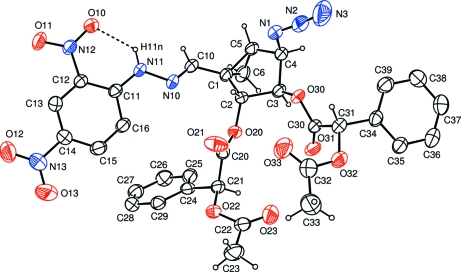
The title compound with numbering scheme used. Hydrogen bonding interactions shown as dotted lines. Ellipsoids are drawn at the 50% probability level. Hydrogen atoms of aromatic-groups have been omitted.

**Fig. 2.**
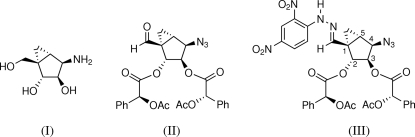
Scheme showing compounds (I), (II) and (III).

## supplementary crystallographic information

### Comment

As part of an ongoing program on the synthesis of furanose ring mimetics (Callam & Lowary, 2000; Callam & Lowary, 2001; Callam *et al.*, 2001; Centrone & Lowary, 2002), we have endeavored to prepare compounds of the general structure (I). The route we developed for the preparation of these materials (Li & Lowary, 2008) started from an achiral starting material and relied upon a late stage resolution by derivatization with *O*-acetyl-(S)-mandelic acid. In the course of synthesizing (I), aldehyde (II) was prepared and, to determine the absolute configuration of the sterogenic centers in the cyclopentane ring, it was reacted with 2,4-dinitrophenylhydrazine to afford hydrazone derivative (III), which is a crystalline solid.

[Insert Scheme 1 here]

The structure of (III) in the crystal is shown in Fig. 1. The five-membered ring adopts an envelope conformation in which C3 is displaced below the plane formed by C1, C2, C4 and C5 and is therefore oriented *cis* to the fused cyclopropane moiety. Thus, the conformation of this ring is similar to that in other bicyclo[3.1.0]hexane analogues (examples: Gurskaya *et al.*, 1990; Gurskaya *et al.*, 1996; Gallucci *et al.*, 2000; Garcia *et al.*, 1992; Guthrie *et al.*, 1981; Màrton-Merész, *et al.*, 1983; Biswas *et al.*, 1996; Bai *et al.*, 2004). The absolute configuration of the stereogenic centers in the molecule could be established by comparison with those present in the (*S*)—*O*-acetylmandeloxyl substituents attached at C2 and C3. Thus, the absolute configuration was established as 1(*R*), 2(*R*), 3(*R*), 4(*R*), 5(S).

[Insert Figure 1 here]

### Experimental

Compound (II) (5.8 mg, 0.011 mmol) was dissolved in CH_3_OH (1 ml) and 1*M* 2,4-dinitrophenylhydrizine (1 ml) was added. The mixture was swirled for 1 min and then the solution was concentrated. The resulting residue was purified by chromatography (3:1 hexane-EtOAc) to give (III) (yield 4.2 mg, 53%) as a yellow solid. This material was recrystallized from CH_3_OH to give a crystalline material (m.p. = 331–333 K).

### Refinement

Hydrogen atoms were generated in idealized positions (according to the *sp*^2^ or *sp*^3^ geometries of their parent carbon or nitrogen atoms), and then refined using a riding model with fixed C—H (0.95–1.00 Å) and N—H (0.88 Å) and with *U*_iso_(H) = 120% of the *U*_eq_ for the parent atoms.

### Crystal data

**Table:**

C_33_H_29_N_7_O_12_	*F*_000_ = 744
*M**_r_* = 715.63	*D*_x_ = 1.441 Mg m^−^^3^
Monoclinic, *P*2_1_	Melting point: 333 K
Hall symbol: P 2yb	Mo *K*α radiation λ = 0.71073 Å
*a* = 6.8522 (11) Å	Cell parameters from 4802 reflections
*b* = 17.747 (3) Å	θ = 2.3–22.4º
*c* = 13.729 (2) Å	µ = 0.11 mm^−^^1^
β = 99.006 (2)º	*T* = 193 (2) K
*V* = 1648.9 (5) Å^3^	Plate, yellow
*Z* = 2	0.63 × 0.56 × 0.04 mm

### Data collection

**Table:**

Bruker SMART 1000 CCD area-detector/PLATFORM diffractometer	3905 independent reflections
Radiation source: fine-focus sealed tube	3150 reflections with *I* > 2σ(*I*)
Monochromator: graphite	*R*_int_ = 0.040
Detector resolution: 8.192 pixels mm^-1^	θ_max_ = 27.6º
*T* = 193(2) K	θ_min_ = 1.9º
ω scans	*h* = −8→8
Absorption correction: multi-scan(SADABS; Bruker, 2003)	*k* = −22→22
*T*_min_ = 0.757, *T*_max_ = 0.996	*l* = −17→17
13919 measured reflections	

### Refinement

**Table:**

Refinement on *F*^2^	Hydrogen site location: inferred from neighbouring sites
Least-squares matrix: full	H-atom parameters constrained
*R*[*F*^2^ > 2σ(*F*^2^)] = 0.043	*w* = 1/[σ^2^(*F*_o_^2^) + (0.0581*P*)^2^ + 0.2055*P*] where *P* = (*F*_o_^2^ + 2*F*_c_^2^)/3
*wR*(*F*^2^) = 0.104	(Δ/σ)_max_ < 0.001
*S* = 1.04	Δρ_max_ = 0.20 e Å^−^^3^
3905 reflections	Δρ_min_ = −0.23 e Å^−^^3^
471 parameters	Extinction correction: none
1 restraint	Absolute structure: Flack (1983)
Primary atom site location: structure-invariant direct methods	Flack parameter: −0.1 (10)
Secondary atom site location: difference Fourier map	

### Special details

**Table:**

Geometry. All e.s.d.'s (except the e.s.d. in the dihedral angle between two l.s. planes) are estimated using the full covariance matrix. The cell e.s.d.'s are taken into account individually in the estimation of e.s.d.'s in distances, angles and torsion angles; correlations between e.s.d.'s in cell parameters are only used when they are defined by crystal symmetry. An approximate (isotropic) treatment of cell e.s.d.'s is used for estimating e.s.d.'s involving l.s. planes.
Refinement. Refinement of *F*^2^ against ALL reflections. The weighted *R*-factor *wR* and goodness of fit *S* are based on *F*^2^, conventional *R*-factors *R* are based on *F*, with *F* set to zero for negative *F*^2^. The threshold expression of *F*^2^ > 2σ(*F*^2^) is used only for calculating *R*-factors(gt) *etc*. and is not relevant to the choice of reflections for refinement. *R*-factors based on *F*^2^ are statistically about twice as large as those based on *F*, and *R*- factors based on ALL data will be even larger.

### Fractional atomic coordinates and isotropic or equivalent isotropic displacement parameters (Å^2^)

**Table:**

	*x*	*y*	*z*	*U*_iso_*/*U*_eq_	
O10	0.0216 (3)	−0.17710 (12)	0.26418 (16)	0.0430 (5)	
O11	0.0112 (4)	−0.21885 (13)	0.11642 (18)	0.0552 (7)	
O12	0.1584 (4)	−0.05771 (17)	−0.14093 (19)	0.0633 (7)	
O13	0.1090 (9)	0.0610 (2)	−0.1281 (3)	0.1188 (17)	
O20	−0.0140 (3)	0.20972 (11)	0.40781 (13)	0.0325 (4)	
O21	0.2602 (4)	0.20436 (16)	0.33755 (19)	0.0563 (7)	
O22	0.1282 (3)	0.31877 (12)	0.21350 (14)	0.0395 (5)	
O23	0.2147 (4)	0.40018 (15)	0.33712 (19)	0.0561 (7)	
O30	0.2856 (3)	0.23219 (11)	0.60946 (15)	0.0370 (5)	
O31	0.1682 (3)	0.34574 (11)	0.55501 (17)	0.0423 (5)	
O32	0.5724 (3)	0.38917 (12)	0.57755 (17)	0.0437 (5)	
O33	0.6065 (5)	0.29640 (16)	0.4718 (2)	0.0641 (7)	
N1	0.2676 (5)	0.08960 (15)	0.6739 (2)	0.0462 (7)	
N2	0.3933 (6)	0.10936 (17)	0.7397 (3)	0.0646 (9)	
N3	0.5225 (8)	0.1213 (3)	0.7995 (4)	0.113 (2)	
N10	−0.0001 (4)	0.03342 (13)	0.35238 (18)	0.0331 (5)	
N11	0.0016 (4)	−0.03229 (13)	0.29748 (18)	0.0326 (5)	
H11N	−0.0152	−0.0764	0.3242	0.039*	
N12	0.0236 (4)	−0.16687 (14)	0.17555 (18)	0.0351 (6)	
N13	0.1177 (5)	−0.00261 (19)	−0.0941 (2)	0.0532 (8)	
C1	−0.0749 (4)	0.09361 (16)	0.4950 (2)	0.0330 (6)	
C2	0.0664 (4)	0.15750 (15)	0.4847 (2)	0.0310 (6)	
H2	0.1955	0.1370	0.4711	0.037*	
C3	0.0947 (4)	0.19714 (16)	0.5849 (2)	0.0329 (6)	
H3	−0.0124	0.2351	0.5871	0.040*	
C4	0.0799 (5)	0.13409 (17)	0.6595 (2)	0.0399 (7)	
H4	0.0440	0.1531	0.7229	0.048*	
C5	−0.0736 (5)	0.08206 (17)	0.6058 (2)	0.0407 (7)	
H5	−0.0795	0.0293	0.6307	0.049*	
C6	−0.2535 (5)	0.11335 (18)	0.5436 (2)	0.0434 (8)	
H6A	−0.2833	0.1674	0.5514	0.052*	
H6B	−0.3710	0.0803	0.5296	0.052*	
C10	−0.0820 (4)	0.02899 (16)	0.4284 (2)	0.0337 (6)	
H10	−0.1478	−0.0160	0.4423	0.040*	
C11	0.0292 (4)	−0.02746 (16)	0.2031 (2)	0.0295 (6)	
C12	0.0412 (4)	−0.09040 (15)	0.1406 (2)	0.0297 (6)	
C13	0.0683 (4)	−0.08305 (17)	0.0436 (2)	0.0333 (6)	
H13	0.0770	−0.1262	0.0036	0.040*	
C14	0.0826 (5)	−0.01172 (18)	0.0063 (2)	0.0376 (7)	
C15	0.0666 (5)	0.05230 (19)	0.0633 (2)	0.0404 (7)	
H15	0.0730	0.1011	0.0354	0.048*	
C16	0.0419 (5)	0.04441 (17)	0.1591 (2)	0.0367 (7)	
H16	0.0329	0.0883	0.1978	0.044*	
C20	0.0997 (4)	0.22849 (16)	0.3412 (2)	0.0326 (6)	
C21	−0.0100 (5)	0.28412 (16)	0.2676 (2)	0.0343 (6)	
H21	−0.0728	0.3238	0.3042	0.041*	
C22	0.2309 (5)	0.37810 (18)	0.2566 (2)	0.0410 (7)	
C23	0.3617 (6)	0.4100 (2)	0.1896 (3)	0.0552 (9)	
H23A	0.4046	0.4606	0.2119	0.066*	
H23B	0.2888	0.4127	0.1223	0.066*	
H23C	0.4776	0.3775	0.1904	0.066*	
C24	−0.1682 (4)	0.24595 (16)	0.1939 (2)	0.0340 (6)	
C25	−0.3494 (5)	0.22742 (18)	0.2210 (2)	0.0410 (7)	
H25	−0.3730	0.2374	0.2861	0.049*	
C26	−0.4943 (5)	0.1948 (2)	0.1538 (3)	0.0472 (8)	
H26	−0.6183	0.1830	0.1727	0.057*	
C27	−0.4619 (5)	0.17881 (19)	0.0590 (3)	0.0445 (8)	
H27	−0.5626	0.1557	0.0133	0.053*	
C28	−0.2813 (5)	0.19683 (18)	0.0312 (2)	0.0401 (7)	
H28	−0.2579	0.1863	−0.0338	0.048*	
C29	−0.1357 (5)	0.23022 (17)	0.0986 (2)	0.0361 (6)	
H29	−0.0121	0.2425	0.0795	0.043*	
C30	0.2988 (5)	0.30679 (16)	0.5946 (2)	0.0336 (6)	
C31	0.5036 (5)	0.33491 (17)	0.6404 (2)	0.0370 (6)	
H31	0.5980	0.2916	0.6511	0.044*	
C32	0.6236 (5)	0.3619 (2)	0.4925 (3)	0.0455 (8)	
C33	0.7038 (6)	0.4211 (3)	0.4342 (3)	0.0609 (10)	
H33A	0.8478	0.4161	0.4416	0.073*	
H33B	0.6699	0.4708	0.4577	0.073*	
H33C	0.6469	0.4156	0.3645	0.073*	
C34	0.4884 (4)	0.37238 (17)	0.7377 (2)	0.0346 (6)	
C35	0.4782 (4)	0.45038 (18)	0.7455 (2)	0.0385 (7)	
H35	0.4847	0.4813	0.6896	0.046*	
C36	0.4582 (5)	0.48276 (19)	0.8356 (3)	0.0438 (8)	
H36	0.4517	0.5360	0.8409	0.053*	
C37	0.4477 (5)	0.4391 (2)	0.9167 (3)	0.0469 (8)	
H37	0.4335	0.4620	0.9777	0.056*	
C38	0.4579 (5)	0.3617 (2)	0.9095 (2)	0.0471 (8)	
H38	0.4510	0.3312	0.9657	0.056*	
C39	0.4780 (5)	0.3286 (2)	0.8205 (2)	0.0436 (7)	
H39	0.4847	0.2753	0.8160	0.052*	

### Atomic displacement parameters (Å^2^)

**Table:**

	*U*^11^	*U*^22^	*U*^33^	*U*^12^	*U*^13^	*U*^23^
O10	0.0628 (14)	0.0296 (11)	0.0390 (12)	−0.0005 (10)	0.0153 (10)	0.0019 (9)
O11	0.0884 (19)	0.0289 (12)	0.0530 (14)	−0.0069 (12)	0.0262 (13)	−0.0121 (11)
O12	0.088 (2)	0.0608 (17)	0.0464 (14)	−0.0106 (15)	0.0278 (14)	−0.0110 (13)
O13	0.241 (6)	0.064 (2)	0.067 (2)	0.016 (3)	0.072 (3)	0.0218 (18)
O20	0.0412 (11)	0.0278 (10)	0.0303 (10)	−0.0004 (8)	0.0112 (8)	0.0030 (8)
O21	0.0488 (14)	0.0656 (16)	0.0589 (15)	0.0116 (12)	0.0221 (11)	0.0270 (13)
O22	0.0535 (13)	0.0313 (11)	0.0355 (11)	−0.0096 (10)	0.0122 (9)	0.0032 (9)
O23	0.0676 (16)	0.0490 (15)	0.0513 (16)	−0.0142 (13)	0.0082 (12)	−0.0091 (12)
O30	0.0493 (12)	0.0194 (9)	0.0408 (11)	−0.0041 (9)	0.0023 (9)	−0.0014 (9)
O31	0.0510 (13)	0.0247 (10)	0.0493 (13)	−0.0023 (9)	0.0019 (10)	0.0030 (9)
O32	0.0540 (13)	0.0324 (11)	0.0459 (13)	−0.0088 (10)	0.0114 (10)	−0.0019 (10)
O33	0.089 (2)	0.0449 (15)	0.0640 (16)	0.0043 (14)	0.0289 (14)	−0.0105 (13)
N1	0.0712 (19)	0.0270 (13)	0.0392 (15)	0.0008 (13)	0.0046 (14)	0.0003 (12)
N2	0.092 (3)	0.0345 (17)	0.059 (2)	0.0112 (17)	−0.0134 (19)	−0.0037 (15)
N3	0.132 (4)	0.069 (3)	0.111 (4)	0.031 (3)	−0.063 (3)	−0.034 (3)
N10	0.0405 (13)	0.0231 (12)	0.0352 (13)	−0.0021 (10)	0.0050 (11)	−0.0063 (10)
N11	0.0425 (14)	0.0209 (11)	0.0341 (13)	−0.0011 (10)	0.0053 (10)	−0.0039 (10)
N12	0.0409 (14)	0.0265 (12)	0.0394 (14)	−0.0003 (10)	0.0112 (11)	−0.0030 (11)
N13	0.066 (2)	0.0556 (19)	0.0403 (16)	−0.0009 (15)	0.0160 (14)	0.0046 (15)
C1	0.0434 (16)	0.0249 (13)	0.0321 (15)	−0.0051 (12)	0.0108 (12)	−0.0023 (12)
C2	0.0443 (16)	0.0216 (13)	0.0278 (14)	0.0002 (12)	0.0077 (12)	0.0019 (11)
C3	0.0413 (16)	0.0223 (13)	0.0362 (16)	−0.0034 (12)	0.0091 (12)	−0.0012 (12)
C4	0.065 (2)	0.0263 (15)	0.0296 (15)	−0.0030 (13)	0.0107 (14)	−0.0010 (12)
C5	0.066 (2)	0.0237 (14)	0.0350 (16)	−0.0091 (14)	0.0176 (15)	0.0009 (12)
C6	0.0493 (19)	0.0332 (16)	0.053 (2)	−0.0090 (14)	0.0240 (15)	−0.0072 (15)
C10	0.0404 (16)	0.0223 (13)	0.0382 (16)	−0.0037 (11)	0.0062 (13)	−0.0018 (12)
C11	0.0275 (13)	0.0261 (13)	0.0341 (15)	0.0015 (11)	0.0021 (11)	−0.0008 (11)
C12	0.0305 (14)	0.0250 (13)	0.0331 (15)	−0.0008 (11)	0.0037 (11)	−0.0004 (12)
C13	0.0330 (15)	0.0333 (15)	0.0339 (15)	−0.0007 (12)	0.0067 (12)	−0.0030 (13)
C14	0.0411 (16)	0.0391 (17)	0.0322 (15)	0.0008 (13)	0.0048 (12)	0.0017 (13)
C15	0.0468 (18)	0.0307 (15)	0.0448 (18)	0.0031 (13)	0.0104 (14)	0.0058 (13)
C16	0.0435 (17)	0.0255 (14)	0.0418 (17)	0.0018 (12)	0.0094 (13)	−0.0017 (13)
C20	0.0396 (16)	0.0276 (14)	0.0318 (14)	−0.0051 (12)	0.0096 (12)	−0.0025 (12)
C21	0.0445 (17)	0.0277 (14)	0.0326 (15)	−0.0010 (12)	0.0126 (12)	0.0012 (12)
C22	0.0500 (19)	0.0319 (16)	0.0393 (18)	−0.0047 (13)	0.0011 (14)	0.0069 (14)
C23	0.062 (2)	0.045 (2)	0.058 (2)	−0.0187 (18)	0.0048 (17)	0.0085 (17)
C24	0.0421 (16)	0.0267 (14)	0.0338 (15)	0.0046 (12)	0.0079 (12)	0.0058 (12)
C25	0.0489 (18)	0.0338 (16)	0.0430 (17)	−0.0012 (14)	0.0161 (14)	−0.0014 (14)
C26	0.0440 (18)	0.0454 (19)	0.055 (2)	−0.0042 (15)	0.0144 (15)	0.0017 (16)
C27	0.0469 (18)	0.0374 (18)	0.0470 (19)	−0.0032 (14)	0.0008 (15)	0.0013 (14)
C28	0.0515 (18)	0.0364 (16)	0.0330 (16)	0.0030 (14)	0.0082 (13)	0.0015 (13)
C29	0.0407 (16)	0.0312 (15)	0.0379 (16)	0.0055 (13)	0.0113 (13)	0.0031 (13)
C30	0.0483 (17)	0.0229 (13)	0.0305 (14)	−0.0036 (12)	0.0088 (13)	−0.0011 (11)
C31	0.0452 (17)	0.0263 (14)	0.0390 (16)	−0.0028 (13)	0.0052 (13)	−0.0023 (12)
C32	0.0401 (17)	0.049 (2)	0.0479 (19)	0.0014 (15)	0.0085 (14)	−0.0049 (16)
C33	0.061 (2)	0.069 (3)	0.054 (2)	−0.022 (2)	0.0111 (18)	0.001 (2)
C34	0.0320 (15)	0.0301 (15)	0.0396 (16)	−0.0031 (12)	−0.0006 (12)	−0.0059 (13)
C35	0.0363 (16)	0.0314 (15)	0.0455 (17)	0.0014 (12)	−0.0010 (13)	−0.0038 (14)
C36	0.0346 (16)	0.0370 (17)	0.058 (2)	0.0014 (13)	0.0010 (14)	−0.0144 (16)
C37	0.0379 (17)	0.053 (2)	0.049 (2)	−0.0022 (15)	0.0040 (14)	−0.0137 (17)
C38	0.0462 (18)	0.053 (2)	0.0410 (18)	−0.0026 (16)	0.0047 (14)	0.0025 (16)
C39	0.0464 (18)	0.0339 (15)	0.0499 (19)	−0.0046 (14)	0.0056 (15)	0.0000 (15)

### Geometric parameters (Å, °)

**Table:**

O10—N12	1.232 (3)	C13—C14	1.375 (4)
O11—N12	1.223 (3)	C13—H13	0.9500
O12—N13	1.226 (4)	C14—C15	1.393 (5)
O13—N13	1.220 (5)	C15—C16	1.360 (4)
O20—C2	1.447 (3)	C15—H15	0.9500
O20—C20	1.334 (3)	C16—H16	0.9500
O21—C20	1.189 (4)	C20—C21	1.524 (4)
O22—C21	1.431 (3)	C21—C24	1.521 (4)
O22—C22	1.351 (4)	C21—H21	1.0000
O23—C22	1.194 (4)	C22—C23	1.493 (5)
O30—C3	1.440 (3)	C23—H23A	0.9800
O30—C30	1.345 (3)	C23—H23B	0.9800
O31—C30	1.192 (4)	C23—H23C	0.9800
O32—C32	1.360 (4)	C24—C25	1.390 (4)
O32—C31	1.422 (4)	C24—C29	1.389 (4)
O33—C32	1.198 (4)	C25—C26	1.373 (5)
N1—N2	1.199 (4)	C25—H25	0.9500
N1—C4	1.496 (4)	C26—C27	1.383 (5)
N2—N3	1.130 (5)	C26—H26	0.9500
N10—N11	1.390 (3)	C27—C28	1.388 (5)
N10—C10	1.263 (4)	C27—H27	0.9500
N11—C11	1.341 (4)	C28—C29	1.384 (4)
N11—H11N	0.8800	C28—H28	0.9500
N12—C12	1.451 (4)	C29—H29	0.9500
N13—C14	1.446 (4)	C30—C31	1.529 (4)
C1—C2	1.512 (4)	C31—C34	1.510 (4)
C1—C5	1.534 (4)	C31—H31	1.0000
C1—C6	1.523 (4)	C32—C33	1.478 (5)
C1—C10	1.462 (4)	C33—H33A	0.9800
C2—C3	1.530 (4)	C33—H33B	0.9800
C2—H2	1.0000	C33—H33C	0.9800
C3—C4	1.531 (4)	C34—C35	1.391 (4)
C3—H3	1.0000	C34—C39	1.388 (5)
C4—C5	1.503 (5)	C35—C36	1.390 (5)
C4—H4	1.0000	C35—H35	0.9500
C5—C6	1.492 (5)	C36—C37	1.367 (5)
C5—H5	1.0000	C36—H36	0.9500
C6—H6A	0.9900	C37—C38	1.380 (5)
C6—H6B	0.9900	C37—H37	0.9500
C10—H10	0.9500	C38—C39	1.381 (5)
C11—C12	1.419 (4)	C38—H38	0.9500
C11—C16	1.420 (4)	C39—H39	0.9500
C12—C13	1.379 (4)		
			
C2—O20—C20	117.6 (2)	O20—C20—O21	125.6 (3)
C21—O22—C22	116.7 (2)	O20—C20—C21	109.6 (2)
C3—O30—C30	118.0 (2)	O21—C20—C21	124.7 (3)
C32—O32—C31	115.8 (3)	O22—C21—C20	108.9 (2)
N2—N1—C4	116.5 (3)	O22—C21—C24	107.8 (2)
N1—N2—N3	172.6 (4)	O22—C21—H21	109.3
N11—N10—C10	116.0 (2)	C20—C21—C24	112.3 (2)
N10—N11—C11	119.0 (2)	C20—C21—H21	109.3
N10—N11—H11N	120.5	C24—C21—H21	109.3
C11—N11—H11N	120.5	O22—C22—O23	123.0 (3)
O10—N12—O11	122.3 (3)	O22—C22—C23	110.3 (3)
O10—N12—C12	118.6 (2)	O23—C22—C23	126.8 (3)
O11—N12—C12	119.1 (2)	C22—C23—H23A	109.5
O12—N13—O13	122.7 (3)	C22—C23—H23B	109.5
O12—N13—C14	119.8 (3)	C22—C23—H23C	109.5
O13—N13—C14	117.6 (3)	H23A—C23—H23B	109.5
C2—C1—C5	106.8 (2)	H23A—C23—H23C	109.5
C2—C1—C6	116.0 (2)	H23B—C23—H23C	109.5
C2—C1—C10	119.3 (2)	C21—C24—C25	120.0 (3)
C5—C1—C6	58.4 (2)	C21—C24—C29	120.9 (3)
C5—C1—C10	120.6 (3)	C25—C24—C29	119.1 (3)
C6—C1—C10	120.2 (3)	C24—C25—C26	120.2 (3)
O20—C2—C1	111.8 (2)	C24—C25—H25	119.9
O20—C2—C3	110.0 (2)	C26—C25—H25	119.9
O20—C2—H2	110.1	C25—C26—C27	120.8 (3)
C1—C2—C3	104.7 (2)	C25—C26—H26	119.6
C1—C2—H2	110.1	C27—C26—H26	119.6
C3—C2—H2	110.1	C26—C27—C28	119.5 (3)
O30—C3—C2	113.1 (2)	C26—C27—H27	120.2
O30—C3—C4	108.2 (2)	C28—C27—H27	120.2
O30—C3—H3	110.2	C27—C28—C29	119.7 (3)
C2—C3—C4	104.7 (2)	C27—C28—H28	120.2
C2—C3—H3	110.2	C29—C28—H28	120.2
C4—C3—H3	110.2	C24—C29—C28	120.7 (3)
N1—C4—C3	109.1 (3)	C24—C29—H29	119.7
N1—C4—C5	105.2 (3)	C28—C29—H29	119.7
N1—C4—H4	112.8	O30—C30—O31	125.2 (3)
C3—C4—C5	103.6 (2)	O30—C30—C31	109.8 (3)
C3—C4—H4	112.8	O31—C30—C31	125.0 (3)
C5—C4—H4	112.8	O32—C31—C30	110.0 (2)
C1—C5—C4	107.4 (2)	O32—C31—C34	108.3 (2)
C1—C5—C6	60.4 (2)	O32—C31—H31	109.9
C1—C5—H5	118.0	C30—C31—C34	108.8 (2)
C4—C5—C6	120.2 (3)	C30—C31—H31	109.9
C4—C5—H5	118.0	C34—C31—H31	109.9
C6—C5—H5	118.0	O32—C32—O33	121.4 (3)
C1—C6—C5	61.1 (2)	O32—C32—C33	112.3 (3)
C1—C6—H6A	117.7	O33—C32—C33	126.3 (3)
C1—C6—H6B	117.7	C32—C33—H33A	109.5
C5—C6—H6A	117.7	C32—C33—H33B	109.5
C5—C6—H6B	117.7	C32—C33—H33C	109.5
H6A—C6—H6B	114.8	H33A—C33—H33B	109.5
N10—C10—C1	119.5 (3)	H33A—C33—H33C	109.5
N10—C10—H10	120.3	H33B—C33—H33C	109.5
C1—C10—H10	120.3	C35—C34—C39	119.0 (3)
N11—C11—C12	124.4 (2)	C31—C34—C39	119.8 (3)
N11—C11—C16	119.7 (2)	C31—C34—C35	121.2 (3)
C12—C11—C16	115.9 (2)	C34—C35—C36	119.5 (3)
N12—C12—C11	121.4 (2)	C34—C35—H35	120.2
N12—C12—C13	116.0 (2)	C36—C35—H35	120.2
C11—C12—C13	122.6 (3)	C35—C36—C37	121.0 (3)
C12—C13—C14	118.3 (3)	C35—C36—H36	119.5
C12—C13—H13	120.8	C37—C36—H36	119.5
C14—C13—H13	120.8	C36—C37—C38	119.8 (3)
N13—C14—C13	119.4 (3)	C36—C37—H37	120.1
N13—C14—C15	118.9 (3)	C38—C37—H37	120.1
C13—C14—C15	121.7 (3)	C37—C38—C39	120.0 (3)
C14—C15—C16	119.4 (3)	C37—C38—H38	120.0
C14—C15—H15	120.3	C39—C38—H38	120.0
C16—C15—H15	120.3	C34—C39—C38	120.7 (3)
C11—C16—C15	121.9 (3)	C34—C39—H39	119.6
C11—C16—H16	119.0	C38—C39—H39	119.6
C15—C16—H16	119.0		
			
C20—O20—C2—C1	−130.2 (3)	C2—C3—C4—N1	76.0 (3)
C20—O20—C2—C3	114.0 (3)	C2—C3—C4—C5	−35.7 (3)
C2—O20—C20—O21	2.3 (4)	N1—C4—C5—C1	−89.7 (3)
C2—O20—C20—C21	−179.5 (2)	N1—C4—C5—C6	−155.1 (3)
C22—O22—C21—C24	154.8 (2)	C3—C4—C5—C1	24.9 (3)
C22—O22—C21—C20	−83.2 (3)	C3—C4—C5—C6	−40.5 (4)
C21—O22—C22—O23	2.3 (5)	C4—C5—C6—C1	93.9 (3)
C21—O22—C22—C23	−177.5 (3)	N11—C11—C12—N12	0.1 (4)
C30—O30—C3—C2	99.5 (3)	N11—C11—C12—C13	179.5 (3)
C30—O30—C3—C4	−145.1 (2)	C16—C11—C12—N12	−177.9 (3)
C3—O30—C30—O31	−6.5 (4)	C16—C11—C12—C13	1.5 (4)
C3—O30—C30—C31	170.8 (2)	N11—C11—C16—C15	−178.9 (3)
C32—O32—C31—C34	170.2 (3)	C12—C11—C16—C15	−0.8 (4)
C32—O32—C31—C30	−71.1 (3)	N12—C12—C13—C14	178.9 (3)
C31—O32—C32—O33	1.7 (5)	C11—C12—C13—C14	−0.5 (4)
C31—O32—C32—C33	−176.8 (3)	C12—C13—C14—N13	177.9 (3)
N2—N1—C4—C3	92.0 (4)	C12—C13—C14—C15	−1.2 (4)
N2—N1—C4—C5	−157.4 (3)	N13—C14—C15—C16	−177.3 (3)
C10—N10—N11—C11	159.9 (3)	C13—C14—C15—C16	1.9 (5)
N11—N10—C10—C1	174.7 (3)	C14—C15—C16—C11	−0.8 (5)
N10—N11—C11—C12	177.6 (3)	O20—C20—C21—O22	164.8 (2)
N10—N11—C11—C16	−4.5 (4)	O20—C20—C21—C24	−75.8 (3)
O10—N12—C12—C11	−8.6 (4)	O21—C20—C21—O22	−16.9 (4)
O10—N12—C12—C13	171.9 (3)	O21—C20—C21—C24	102.4 (4)
O11—N12—C12—C11	171.4 (3)	O22—C21—C24—C25	−160.9 (3)
O11—N12—C12—C13	−8.1 (4)	O22—C21—C24—C29	18.1 (4)
O12—N13—C14—C13	−8.9 (5)	C20—C21—C24—C25	79.1 (3)
O12—N13—C14—C15	170.3 (3)	C20—C21—C24—C29	−101.9 (3)
O13—N13—C14—C13	172.5 (4)	C21—C24—C25—C26	178.3 (3)
O13—N13—C14—C15	−8.3 (5)	C29—C24—C25—C26	−0.7 (5)
C5—C1—C2—O20	−136.4 (2)	C24—C25—C26—C27	0.9 (5)
C5—C1—C2—C3	−17.4 (3)	C25—C26—C27—C28	−0.7 (5)
C6—C1—C2—O20	−73.9 (3)	C26—C27—C28—C29	0.3 (5)
C6—C1—C2—C3	45.1 (3)	C27—C28—C29—C24	0.0 (4)
C10—C1—C2—O20	82.4 (3)	C25—C24—C29—C28	0.2 (4)
C10—C1—C2—C3	−158.5 (3)	C21—C24—C29—C28	−178.7 (3)
C2—C1—C5—C4	−4.8 (3)	O30—C30—C31—O32	140.2 (2)
C2—C1—C5—C6	110.6 (3)	O30—C30—C31—C34	−101.3 (3)
C6—C1—C5—C4	−115.4 (3)	O31—C30—C31—O32	−42.4 (4)
C10—C1—C5—C4	135.7 (3)	O31—C30—C31—C34	76.0 (4)
C10—C1—C5—C6	−108.9 (3)	O32—C31—C34—C35	19.9 (4)
C2—C1—C6—C5	−94.4 (3)	O32—C31—C34—C39	−162.3 (3)
C10—C1—C6—C5	109.5 (3)	C30—C31—C34—C35	−99.6 (3)
C2—C1—C10—N10	−13.5 (4)	C30—C31—C34—C39	78.2 (3)
C5—C1—C10—N10	−149.2 (3)	C31—C34—C35—C36	178.0 (3)
C6—C1—C10—N10	141.9 (3)	C39—C34—C35—C36	0.2 (4)
O20—C2—C3—O30	−89.3 (3)	C31—C34—C39—C38	−178.0 (3)
O20—C2—C3—C4	153.2 (2)	C35—C34—C39—C38	−0.1 (5)
C1—C2—C3—O30	150.5 (2)	C34—C35—C36—C37	−0.3 (5)
C1—C2—C3—C4	32.9 (3)	C35—C36—C37—C38	0.3 (5)
O30—C3—C4—N1	−44.9 (3)	C36—C37—C38—C39	−0.2 (5)
O30—C3—C4—C5	−156.5 (2)	C37—C38—C39—C34	0.1 (5)

### Hydrogen-bond geometry (Å, °)

**Table:**

*D*—H···*A*	*D*—H	H···*A*	*D*···*A*	*D*—H···*A*
N11—H11N···O10	0.88	2.00	2.618 (3)	126
